# Communicating with mismatch and tension: treatment provision experiences of primary care doctors treating patients with overactive bladder in Hong Kong

**DOI:** 10.1186/s12875-015-0380-0

**Published:** 2015-10-30

**Authors:** Judy Yuen-man Siu

**Affiliations:** David C. Lam Institute for East-West Studies (Environment, Health, and Sustainability working group), Hong Kong Baptist University, Kowloon Tong, KLN Hong Kong

**Keywords:** Hong Kong, Treatment provision experiences, Primary care doctors, Overactive bladder patients

## Abstract

**Background:**

Overactive bladder (OAB) is a common chronic bladder dysfunction worldwide. As the first contact point of health care, primary health care providers are often consulted by patients seeking initial consultation for OAB. The relatively short history of the existence of OAB in medicine and low public awareness of OAB in Hong Kong, however, often serve as a challenge to primary health care providers in treating patients with OAB. The experiences of patients and health care providers are often influenced by the interaction between these two groups, hence both health care providers and patients are key determinants of the entire treatment experience, and the perspectives of health care providers should not be overlooked. However, patient experiences have been the main focus of related studies, few of which have examined the treatment provision experiences and perspectives of health care providers. This research gap is notable considering that the satisfaction and morale of health care providers can influence treatment outcome.

**Methods:**

This study adopted a qualitative research approach by conducting semistructured individual interviews with 30 private practice primary care doctors in Hong Kong between November 2013 and May 2014.

**Results:**

Lacking confidence in treating OAB patients, encountering mismatch with patients in treatment expectations and communication style, and feeling embarrassed when communicating with OAB patients were the experiences reported by the sampled doctors.

**Conclusion:**

The sampled doctors’ treatment provision experiences revealed a general lack of knowledge about OAB among primary care doctors in Hong Kong. Furthermore, the negative stereotype of and lack of trust in private practice doctors created tension between the doctors and patients. This lack of mutual trust was particularly unfavourable for the doctors to provide long-term treatment and support to patients with OAB. The embedded distrust of private practice doctors also affected the prescribing behaviour of the doctors, who prescribed medication only to satisfy patient demands, which may lead to antibiotic abuse and resistance. Finally, the expectations of doctor professionalism and behaviour in Chinese cultures and the cultural perceptions of urinary diseases caused challenging treatment provision experiences for the sampled doctors.

## Background

Overactive bladder (OAB) is a common chronic bladder dysfunction worldwide. Population-based studies in Europe and Canada have estimated that the overall prevalence of OAB in people aged 40 years or older at 16.6 % [1] and 13.9 % [2] respectively. In Hong Kong, a study estimated that approximately 15 % of the population experienced OAB symptoms [3]. Urinary urgency accompanied by frequency, with or without urgency urinary incontinence, and with the absence of urinary tract infection (UTI) or other obvious pathology [4] are defined as OAB. Approaches to treating and managing OAB include lifestyle modifications, behavioural therapy, pharmacotherapy, neuromodulation, botulinum toxin therapy, and surgical intervention [5].

As the first contact point in the health care system, primary health care providers are often consulted by patients who require specialist secondary care [6], and the evaluation and treatment of OAB often starts in primary care doctors’ offices [7]. The American Urological Association recommends in a new guideline on OAB care that primary care doctors be placed in the position of gatekeeper for OAB, in which stable patients are advised to remain under the care of primary care providers [7]. The relatively short history of the existence of OAB in medicine and low public awareness of OAB in Hong Kong, however, often serves as a challenge to primary health care providers in treating patients with OAB.

This study was inspired by earlier research on patients with OAB, covering their illness experiences [8] and doctor-shopping behaviour [9]. Doctor shopping involves patients visiting different doctors every time they are sick rather than consulting the same doctor. These earlier studies showed that sampled OAB patients’ doctor-shopping behaviour was closely related to their unpleasant illness experiences [9], which often started when they first experienced OAB symptoms and sought an initial consultation for them. Their unpleasant experiences had numerous causes. The sampled patients reported difficulties in the treatment-seeking process in addition to physical bladder symptoms. The flippant attitudes of doctors often made these patients feel embarrassed during the treatment process, and the long but inefficacious treatment also made the patients frustrated with the treatment outcome. Furthermore, the patients commonly perceived doctors as lacking the necessary empathy to understand their conditions and difficulties, making their illness experiences unpleasant. Past studies have shown that patients who seek medical treatment for OAB often encounter negative experiences such as experiencing miscommunication with health care providers [10, 11] and receiving treatment from doctors who lack knowledge about the diagnostic tests for OAB [11].

The experiences of patients and health care providers are often influenced by the interaction between these two groups, hence both health care providers and patients are key determinants of the entire treatment experience, and the perspectives of health care providers should not be overlooked. Treatment experience not only influence patient treatment satisfaction, compliance, and thus treatment outcome [12]; it can also affect the satisfaction and morale of health care providers [13] and in turn influences treatment outcome [13]. However, patient experiences have been the main focus of past research. Studies on the treatment provision experiences and perspectives of health care providers, though crucial, are lacking. The literature has a paucity of research on the treatment provision experiences of health care providers in Chinese communities, and even less is known about their treatment provision experiences involving patients with OAB. Therefore, investigating the treatment provision experiences of health care providers treating patients with OAB is crucial for improving the experiences of both parties. Primary health care providers are the first contact of patients, including those with OAB; hence, the experiences of primary health care providers are critical because these experiences can influence patient motivation to seek and follow advice and treatment. In view of the aforementioned literature gap, this study adopted a qualitative approach to investigate the treatment provision experiences of primary health care doctors treating patients with OAB in Hong Kong.

## Methods

To understand the experiences of primary health care doctors treating patients with OAB, a qualitative study approach was used.

### Data collection

In this qualitative study, in-depth individual semistructured interviews were conducted between November 2013 and May 2014 with 30 primary health care doctors who had provided treatment to patients with OAB in Hong Kong. Purposive sampling was used to identify 30 primary health care doctors with experience treating patients with OAB and who satisfied the following inclusion criteria: doctors who (a) were medical practitioners working in private practice primary care, and (b) had treated at least one patient with OAB within the past 3 years. Because private practice primary care doctors constitute the largest proportion of primary health care providers in Hong Kong [14], most patients with OAB can be assumed to initially consult private practice primary care doctors. Hence, this study recruited only primary care doctors working in the private sector.

The sampling of primary care doctors was based on referrals from patients with OAB who were interviewed for an earlier study. These patients were asked to identify the primary health care doctor they consulted first regarding their bladder condition. This approach ensured that the sampled doctors had treated patients with OAB, and it minimised the likelihood of inaccurately sampling primary care doctors who had treated patients with bladder symptoms similar to those of OAB but without a confirmed OAB diagnosis. The qualifications of the identified health care providers were then verified using the website of the Medical Council of Hong Kong. Health care providers who were on the resident list and without specialist registration (except for family medicine) were defined as primary care doctors [15]. These doctors were then sampled, contacted, and invited for the interviews. Thirty nominated doctors were approached in the first attempt, with 21 doctors accepted the interviews, and 9 doctors declined. These doctors who declined the interview were replaced by other primary care doctors referred by the patients (6 doctors were approached and accepted the interviews), and referred by the members of an OAB patient self-help group (3 doctors were approached and accepted the interviews).

Thirty primary care doctors were interviewed individually between November 2013 and May 2014. Although a study documented that 12 interviews are sufficient for data saturation [16], another study have suggested 17 interviews as sufficient [17]. This study conducted 30 interviews to yield a greater confidence in the data. All the interviews were conducted by the researcher to ensure quality and consistency. Doing so reduced the possibility of insufficient data, and avoided variation in data collection being caused by introducing another interviewer. All the interviews were conducted in Cantonese Chinese, the mother tongue of the sampled doctors and the researcher, thus the doctors could express themselves clearly without a language barrier.

An interview question guide was developed prior to the interviews. The interview questions were developed based on earlier studies on the illness experiences of patients with OAB [8] and their doctor-shopping behaviour [9]. Other past studies about the treatment provision experiences of health care providers were consulted to develop the interview question guide. Conducting the interviews with the interview question guide also ensured that the interviews stayed within the scope of the research topics. An open questioning style was adopted with some closed questions to clarify particular points. This allowed the sampled doctors had a high degree of flexibility to express their views, feelings, and experiences [18]. The questions, which were aimed at investigating the understanding of OAB among the sampled primary care doctors, their experiences in dealing with patients with OAB, and their feelings about these patients, included the following:

1. What are the most common complaints (physical and/or emotional) of OAB patients?
2. What are your immediate thoughts about the possible diagnoses of a patient who presents bladder symptoms?
3. Will OAB immediately come to your mind when a patient complains about bladder symptoms?
4. What kinds of examination and treatment will you provide for OAB patients?
5. If all the examination procedures show normal results but bladder symptoms persist in a patient, how will you manage this patient?
6. How do you reach a diagnosis of OAB?
7. If a patient is diagnosed with OAB, how will you proceed?
8. How much do you know about OAB?
9. How confident are you in managing OAB patients? Why?
10.  Can you share some experiences in treating OAB patients?
11.  Do you think there is any difference in communicating with and treating OAB patients and other patients in your clinic? If so, what are these differences?
12.  Have you ever experienced any difficulties in communicating with OAB patients?

To protect the privacy of the sampled doctors, the interviews were conducted either in their clinics or a meeting room at Hong Kong Baptist University. Each interview lasted between 1 and 1.5 h, and was audio-recorded with the consent of the participant. A keychain with the logo of the researcher’s institution was presented to each sampled doctor as a gift to acknowledge their time and sharing after each interview.

### Data analysis

Quick data analysis, involving initial data scanning to determine what information had been obtained and what topics needed to be explored further [19], was conducted by the researcher during the interviews. Interviews were transcribed verbatim by two student assistants, and the sampled doctors were asked to read the transcriptions to ensure accuracy. The transcribed interviews were then translated into English by the researcher, and back-translation was conducted by a bilingual student assistant to ensure that the translated transcripts accurately reflected the originals. The back-translation also assisted in crosschecking the first-round translation and minimised errors arising from potential researcher bias.

The researcher conducted the entire coding and data analysis procedure. Thematic analysis was adopted [20]. Interview transcriptions were segmented into meaning units and then collapsed into categories and eventually themes through abstraction and constant comparison. Coding tables were developed and constructed [20], identifying themes, categories, and codes with supporting interview quotes according to the inductive coding process by allowing the discovery of patterns of behaviour and thoughts [18]. Repetitive codes and themes were noted and highlighted, and new thematic codes that emerged from the data were added to the coding table. Memos were used to record ideas and commentary in the interview and coding process. The analytic procedures, codings, and findings were documented in the codebook to ensure the consistency and accuracy of the data collected. Neutrality of data was achieved, with the direct interview transcripts referenced in the coding and analysis, thus grounding the findings in the interview data. Data saturation was achieved.

### Ethical considerations

Ethical approval was obtained from the Committee on the Use of Human and Animal Subjects in Teaching and Research at Hong Kong Baptist University prior to the study. Participants’ participation in the study was voluntary. Information sheets explaining the purpose and nature of the study, research procedures, and use of the collected data in academic publications were provided to the sampled doctors in their native written language (traditional Chinese). Verbal explanation and clarification was provided to them wherever required. Signed informed consent was obtained from all the interviewees, and they were assured of their rights and freedom to withdraw from the study at any time. To ensure privacy, no identifying details were recorded in the audio records or coded data. All the sampled doctors were designated with codes in the interview transcripts. The data were stored in locked cabinets and treated with strict confidentiality. The audio records of the interviews were destroyed after the interviews were transcribed.

## Results

### Participants

All 30 participants in this study were primary care doctors working in private practice in Hong Kong, and all of them were men aged between 38 and 62 years. Each had worked as a primary care doctor for at least 10 years, and had worked in private practice for at least 7 years. All the sampled doctors had worked in public hospitals, and 13 of them had worked in public general outpatient clinics afterward, prior to entering private primary care practice. Among the 30 doctors, seven had completed specialty training in family medicine, whereas the others had received no specialty training in any field after their general medicine training (i.e., MBBS or MBChB). Of these remaining 23 doctors, nine worked as private practice primary care doctors immediately after finishing their mandatory 1-year public hospital internship. All the sampled doctors had completed a rotation in four disciplines—internal medicine, surgery, paediatrics, and obstetrics and gynaecology—in their internship year.

Visits by patients complaining about urinary and bladder discomfort in the sampled doctors’ clinics were common, and ordering urinalysis was the most frequent response in these cases. Although most of these patients were ultimately diagnosed as having UTI, some of them showed no sign of having any obvious pathogen. For these patients, the sampled doctors would reach a preliminary diagnosis of OAB. Providing treatment to patients with OAB was uncommon according to the sampled doctors, although they noted a steady rise in the number of OAB patient visits to their clinics in recent years. As the initial management plan for these patients whose bladder conditions were not affected by an obvious pathogen, the doctors typically prescribed a course of antibiotics. Referral to a urologist was the next treatment step for patients who returned without improvement. However, making referrals was not always straightforward, according to the doctors; the patients often either did not return to them for follow-up treatment or rejected the referral because of the higher treatment fees for receiving specialised urology treatment.

The sampled doctors generally encountered four types of experiences when treating patients with OAB, namely, lacking confidence in treating OAB patients, experiencing mismatch in treatment expectations with the patients, experiencing mismatch in communication with the patients, and feeling embarrassed when communicating with OAB patients.

### Treatment provision experiences of the sampled primary care doctors treating patients with overactive bladder

#### Insufficient confidence in treating patients with OAB

As primary care doctors without formal urology training, all the sampled doctors reported having a highly limited knowledge of OAB, leading them to lack confidence in treating and caring for patients with OAB. Although the doctors said that they knew about the symptoms of OAB, they did not know exactly how to diagnose OAB or treat patients with the condition, as the following quote reveals:

I learned about the symptoms of OAB in some CME [continuing medical education] courses. However, OAB is a complicated condition and it is quite impossible to confirm in primary care settings. Usually the patient needs specific tests that cannot be done by GPs [general practitioners]. Without the support of these tests, I am not confident in telling these patients about my provisional diagnosis. However, the patients often expect me to give them a definite diagnosis. Honestly, I am not confident in dealing with these patients. Patients cannot understand my limitation as a GP, and I cannot tell them [about these limitations] or they will think that I am incapable. [P4]

Ordering urinalysis was the usual examination procedure adopted by the sampled doctors for the patients complaining about urinary symptoms; however, the doctors often encountered difficulty and uncertainty moving to the next treatment step if the urinalysis results were normal. In addition to satisfying the perceived expectations of patients, prescribing a course of antibiotics was the simplest management plan for such cases. The following quote is representative of the view of many of the doctors:

Ordering urinalysis is the most common procedure for patients who complain about bladder discomfort. It is easy to handle patients who suffer from UTI since just a course of antibiotics can easily satisfy these patients. However, if the patient’s urinalysis shows normal findings, then the real headache comes. Patients often expect medication from doctors. It is only with medication that the patients feel they are receiving treatment. Without medication, they will not think that the consultation is worthwhile. If you do not prescribe any medicine to patients, they will argue with you or not pay the consultation fee; so I usually prescribe a course of antibiotics to make them satisfied, even though I know antibiotics have nothing to do with their condition. [P17]

Because of the difficulty in diagnosing OAB in primary care settings and the uncertainty of the doctors regarding how to treat their patients, the sampled doctors often advised the patients to not be overly concerned regarding their bladder discomfort. However, such advice could lead to the doctors being viewed by their patients as uncaring. One doctor recalled the following:

Very often, when patients come to see a doctor, they assume that they are sick and need treatment. I came across a patient who complained about urinary frequency and urgency. As usual, I ordered urinalysis for her, but the test result showed normal findings without any sign of UTI. I suspected she might have OAB, but I could not confirm with confidence since I had my limitation [as a GP]. Therefore, there was little more that I could do except advise her to relax. However, she was not satisfied, and kept asking me whether she was infected with rare bacteria. I finally gave her a course of antibiotics to satisfy her. By prescribing antibiotics, you can make the patient trust you, although you know that you have limitations in dealing with the patient’s condition. [P21]

#### Mismatch in treatment expectations

OAB is a long-term bladder condition; its chronicity implies that obtaining full recovery is difficult for most patients. However, according to the experiences of the sampled doctors, their patients appeared failing to understand the limitations of OAB treatment. As a result, being blamed by patients for delaying treatment was common among the doctors, as one recalled:

Usually patients expect to be treated quickly when they come to a doctor. If they fail to recover quickly, they blame the doctors either for being incapable or deliberately prolonging treatment. I still remember how a woman patient suffering from urinary urgency and incontinence blamed me; she was dissatisfied because she failed to get better after several rounds of treatment that I had ordered. I told her to have patience, and suggested that I refer her to another specialist for further evaluation and treatment. However, she refused, and accused me of cheating her out of money by prolonging treatment and referring her to another specialist. [P26]

Mismatch in treatment expectations between the sampled doctors and patients occasionally led to tension between them. One doctor shared the following:

I often tell my patients to have patience in dealing with their chronic bladder conditions. However, not many patients can accept my advice. They often expect quick efficacy after a course of medication or two, although such an outcome is ‘mission impossible’ in their cases. I often tell my patients that it is quite impossible for them to fully recover, and that they should expect less efficacious treatment to avoid disappointment since there are still many limitations in the current treatment for their bladder condition. However, they complain, saying that I am not capable or attempting to cheat them for more money by prolonging the treatment. This is particularly the case for newer patients since they still have not yet built up trust in me. [P23]

#### Mismatch in communication style

As experienced by the sampled doctors, there was significant mismatch in expectation about the communication style between themselves and their patients. To the doctors, they perceived their patients as feeling embarrassed during consultations; thus, the sampled doctors adopted a more casual communication style during consultations and treatment in an attempt to ease this embarrassment. However, the sampled doctors perceived their patients interpreting such a casual communication style as demonstrating insincerity and a lack of empathy, confusing these doctors as to an optimal communication style to adopt with their patients. One doctor shared the following:

As most patients feel embarrassed when talking about their bladder symptoms and conditions, I tend to use a more casual tone to communicate with them in order to make them feel more relaxed. However, some patients are offended by this casual consultation style, and some of them ask me if I could stop teasing them. Sometimes I really feel puzzled as to what communication style I should use. I just want them to feel relaxed during the consultation, but it seems that not all patients can accept such a casual style from doctors. [P6]

During the study period, the symptoms of OAB, particularly urinary frequency and urinary incontinence, were identified by government antisubstance abuse public service announcements as signs and consequences of ketamine abuse. These public service announcements created a new stereotype regarding OAB symptoms and thus depicted people with these symptoms as potential ketamine users. The internalisation of this new stereotype influenced the history-taking approach of the sampled doctors with their patients during consultation, which could make these patients feeling offended according to the sampled doctors’ experiences, as the following quote illustrates:

When patients come to my clinic complaining about urinary frequency, urgency, or even incontinence, I ask them whether they have ever engaged in substance abuse in addition to focusing on whether they might have UTI. As you know, ‘little K’ [ketamine] can also lead to similar bladder symptoms, so asking patients if they have such experience has become necessary in the history taking. Some patients indeed feel offended about such a question, particularly those patients who have seen me for many years, and this can ruin the trust between us. This is a dilemma for me, because as a doctor, I have the responsibility to investigate every possibility; however, not many patients understand this. [P24]

Because the symptoms of OAB are similar to those of some sexually transmitted diseases, the sampled doctors had to ask detailed questions about the sexual histories of the patients. However, their patients were often shocked for being asked such questions according to the sampled doctors’ experiences, and the doctors said that such questioning often led to the patients losing trust in them, as indicated in the following quote:

It is necessary and usual for a doctor to know about the sexual life of a patient who complains about urinary problems, because some urinary symptoms are actually symptoms of sexually transmitted diseases. However, some patients are offended when asked such questions, which are often perceived as sensitive and private. They would consider whether I doubt and do not trust them. Once patients are offended, the mutual trust between them and their doctor is broken, and it is difficult to expect cooperation from the patients in later consultations. Even worse, they may never come to you again. [P15]

#### Embarrassment

Urinary problems are commonly perceived as embarrassing in Chinese cultures, and the patients showed embarrassment when seeking treatment from the sampled doctors according to these doctors’ observation. This embarrassment spread to the doctors during consultations, as noted in the following quote:

Doctors are trained to be professional and not show any embarrassment during the physical examination of patients. Once a doctor shows embarrassment, the patient will feel embarrassed, too. However, not feeling embarrassed can be quite difficult to achieve in real practice. Urinary problems are embarrassing because they often involve sensitive issues; the physical location of the urinary system, the patient’s sexual life and habits, etc., all make the situation embarrassing. We have to rule out sexually transmitted diseases since the symptoms of these diseases can be very similar to urinary problems, so we have to ask about the patient’s sexual practices in detail. These are embarrassing topics in Chinese culture; so, honestly, I sometimes feel embarrassed, especially when the patient is embarrassed. [P19]

The sense of embarrassment of these doctors was more severe when they encountered women patients, as illustrated by the following quote:

Asking about urinary problems is particularly embarrassing with women patients, because in most cases, they feel very embarrassed in talking about their bladder symptoms. However, to make a diagnosis, I have to ask about their histories in detail, such as their sex lives and practices, and they feel even more embarrassed after these questions. Some women patients also feel embarrassed with the physical examination. Their embarrassment often makes me feel embarrassed, though I cannot show this in the procedure. If some women patients are really embarrassed, then I may skip asking some very sensitive questions. [P11]

## Discussion

As primary care doctors, those sampled serve as the first contact for most patients, including patients with OAB. However, the sampled doctors experienced challenges when treating patients with OAB. Lacking confidence in treating patients with OAB, experiencing mismatch in both treatment expectations and communication style with OAB patients, and feeling embarrassed when communicating with OAB patients were the experiences encountered by the sampled doctors. The lack of knowledge and support in treating patients with OAB, the embedded tension between private practice doctors and patients in Hong Kong, the expectations of doctor professionalism and behaviour in Chinese cultures, and the cultural perceptions of urinary diseases all made treatment provision challenging for the sampled doctors.

Past studies have shown that primary care doctors often provide little appropriate and accessible information about OAB to patients with the condition [6]. The insufficient knowledge about OAB and lack of diagnostic instruments in primary care settings often limited the confidence of the sampled doctors in diagnosing and treating patients with OAB. However, the common patient expectations of receiving a confirmed diagnosis often presented the sampled doctors with a dilemma. The doctors were aware of their limitations regarding knowledge and the tests available for potential OAB patients; but were reluctant to reveal these limitations to patients because doing so may have subverted patient expectations. Addressing their limitations in diagnosing procedures and treatment provision to patients was perceived as taboo by the sampled doctors, who said that doing so would damage the patients’ trust in the capability of doctors. Doctors represent the authority of medical knowledge; in Chinese culture, patients in particular have high regard for their doctors’ recommendations [21], and doctors’ treatment decisions are respected and trusted [22]. Therefore, receiving respect and deference from public is expected by doctors in Chinese culture [22, 23]. At the same time, patients also expect doctors to be highly capable in confirming diagnoses and providing effective treatment in Chinese culture. These popular attitudes and expectations regarding doctors contributed to the reluctance of the sampled doctors, who did not wish to ‘lose face’, to articulate their limitations to their patients.

The high social status and respect accorded in the Chinese cultures can also result in arrogance among doctors [24], making the medical virtue of humility difficult to understand and practice [25], which could ultimately affect the sampled doctors’ treatment provision experiences. The practice of humility as a medical virtue encompassing unpretentious openness, honest self-disclosure, the avoidance of arrogance, and the modulation of self-interest, though valued, can also indicate doctor weakness and indecisiveness [25], implying incapability and thus putting the doctor at risk of losing face. Some sampled doctors reported being perceived and blamed as incapable by their patients because of their limitations in making definite diagnoses of OAB and providing treatment in primary care settings. The intention of the sampled doctors to protect their professional image and avoid breaking the taboo of admitting their limitations, thus, may have exacerbated the tension between the doctors and patients, making the treatment provision experiences unpleasant for the sampled doctors. Thus, both the sampled doctors and their patients were disadvantaged in the treatment process.

Coordinating care for patients by making referrals to secondary health care specialists for further treatment is a major role of primary care doctors [26]. However, coordination between primary and secondary health care providers is not always straightforward [6], especially in places like Hong Kong where patients do not have high trust or confidence in private practice doctors, and where private practice doctors are often perceived as profit-oriented [27]. As this study demonstrated, making referrals could be perceived by some patients as a deceptive money-making technique because of embedded negative stereotypes about and lack of trust in private practice doctors in Hong Kong. Therefore, any additional procedures and referrals made by private practice doctors have a high chance of being interpreted by patients as attempts to extract more money from them. As a result of such cultural stereotypes of private practice doctors, the sampled doctors reported being accused of intentionally delaying treatment and seeking more money by making referrals to secondary specialists. As a result, the sampled doctors were reluctant to make such referrals, even though they recognised their limitations in treating patients with OAB. Their treatment provision experiences were thus affected.

The differing definition of ‘treatment’ between the sampled doctors and their OAB patients often led to tension in the doctor–patient relationship. To the sampled doctors, the consultation and physical and pathological examination procedures were considered ‘treatment’ even though no medication was prescribed. However, their patients, who considered themselves to be sick, viewed medication as an integral component of the entire treatment process, according to the doctors. Unlike patients in Western countries, among whom expecting prescriptions is less common [28], patients in Hong Kong typically expect doctors to prescribe medication after a consultation [29]. Only after being prescribed medication do most patients consider consultation and treatment as complete. According to the sampled doctors, when the expectation of being prescribed medication was not met, patients became dissatisfied. Thus, the doctors felt forced to prescribe medication to satisfy the patients, which many of them did. Such difference in the definition of ‘treatment’ between the sampled doctors and their patients resulted in tension between them. The sampled doctors also said that the feeling of being pressured to prescribe medication created an unpleasant experience in the treatment provision process.

Although patient expectations played a substantial role in contributing to the cultural definition of ‘treatment,’ the sampled doctors also played a crucial role in reinforcing the view that prescribing medication was a necessary part of treatment. Bladder problems were widely perceived as being caused by bacterial infections among the sampled doctors’ patients, who thus viewed antibiotics as the ideal treatment. To meet the patients’ expectations, some sampled doctors prescribed antibiotics even though they knew that doing so would yield no improvement. Indeed, doctor perceptions of patient expectations for antibiotics are strong factors in antibiotics prescription [30]. Antibiotic efficacy reaches near-mythical levels among patients [31], and doctors frequently prescribe antibiotics to err on the side of caution or satisfy the patient expectations [32–34], leading antibiotics to become overprescribed worldwide [31]. Antibiotics, thus, were an effective medium through which the sampled doctors were able to satisfy their patients, which for them was crucial considering existing doctor–patient tension and the culture of mistrust in private practice doctors in Hong Kong. The prescription of antibiotics also demonstrated the sampled doctors’ lack of confidence in handling OAB patients because of their limited knowledge and diagnostic testing ability; thus the doctors were motivated to prescribe antibiotics, which they expected their patients to desire. Prescribing antibiotics was viewed by the sampled doctors as their only choice because doing so could fulfil patient demands and maintain their professional dignity and perceived capability. However, not only did prescribing antibiotics delay OAB patients receiving appropriate treatment, more importantly it resulted in the misuse and abuse of antibiotics, leading to antibiotic resistance that can endanger public health [31].

Differing expectations of bladder condition treatment and outcome between the sampled doctors and their patients also created tension in the doctor–patient relationship, thus making the treatment provision experiences unpleasant. OAB is a chronic condition, therefore full recovery was considered impossible by the sampled doctors. However, the patients often expected quick efficacy or even a cure. With such differences in treatment outcome expectation, in addition to the embedded distrust and negative stereotypes of private practice doctors in Hong Kong, the sampled doctors reported being blamed and perceived as having intentionally prolonged treatment to extract more money from their patients. The tension between doctors and patients increased further if there was a lack of communication about the gap between the actual situation and patient expectations regarding treatment outcomes, thus making the treatment provision experiences even more unpleasant for sampled doctors. Patients with OAB should be informed about the limitations of their current treatment and advised to think realistically regarding the treatment outcome; this would not only help the patients cope with the condition more positively, but also improve the treatment provision experience and morale of health care providers.

Mismatch in communication style between the sampled doctors and their patients also created tension between them, leading to unpleasant treatment provision experiences. Patients in Chinese cultures tend to have a high respect for doctors [21]. Stereotypes of doctors’ authority and professionalism also prevail, reinforcing the hierarchical differential [35]. In the current study, such stereotypes, in addition to the perceived higher hierarchical status of doctors, influenced not only how patients communicated with the sampled doctors, but also the patient expectations of doctor communication style. The doctors were expected to communicate in a serious and professional manner, and show empathy for the suffering of their patients. The casual and relaxed communication style adopted by the doctors violated the expectations of the patients, who interpreted the style as indicating that the doctors were insincere and lacked empathy. Miscommunication resulted from the mismatch in communication style between the sampled doctors and their patients, thus the doctor–patient tension increased further, worsening the treatment provision experiences of the doctors as a result.

Diseases are often attached with stigma [36]. The literature shows that not only do the patients with stigmatised diseases endure painful experiences [37], but the health care providers also encounter difficult experiences when treating them [38]. Disease stigmas can influence the communication between treatment providers and patients because both of them have internalised the stigmas [39], making the communication between them appear judgemental to the other. The urinary symptoms of OAB are similar to those of sexually transmitted diseases and ketamine use, both of which are severely stigmatised in Hong Kong. Therefore, patients with OAB are highly vulnerable to having their conditions misinterpreted. Because of the entrenched stigma of sexually transmitted diseases and the new stereotyping of bladder symptoms as being a consequence of ketamine use, the internalisation of these stigmas further intensified the communication tension and mismatch between the sampled doctors and their patients. Although the sampled doctors did not intend to stigmatise the OAB patients as potential sexually transmitted disease carriers or ketamine users, and though it is a clinical norm for doctors to investigate their patients’ personal habits and health histories, history taking involving sensitive topics such as sexual practices and ketamine use could easily have made the patients feel like they were being stereotyped by the doctors. This could damage the mutual trust between the sampled doctors and their patients, worsening the doctor–patient relationship. This could also affect the treatment satisfaction of the patients (and subsequently the treatment compliance and outcomes) as well as contribute to unpleasant treatment provision experiences of the sampled doctors. However, none of the sampled doctors exhibited awareness of the importance of clarifying with patients the reasons for asking the sensitive questions. Thus, the mutual misunderstanding between the sampled doctors and their OAB patients remained unresolved, unfavourably affecting their long-term treatment relationship.

Because of the characteristics and stigmas of OAB, patients often felt embarrassed when they communicated about their bladder symptoms with the sampled doctors. The gender difference between the sampled doctors and their patients often compounded the overall embarrassment. The patients’ embarrassment frequently spread to the doctors, even though such a sense of embarrassment violated the expectation of doctors to maintain a high level of professionalism. As noted by some of the sampled doctors, a sense of embarrassment affected their consultation and history-taking practices by leading them to avoid asking sensitive questions, which could have hindered them from diagnosing appropriately and thus affected the treatment outcome.

### Looking ahead: The possibility of primary care doctors to serve as gatekeepers in OAB care in Hong Kong

Differences in the explanatory models between doctors and patients about a disease can negatively influence doctor-patient relationship [40], and thus the treatment provision experiences of doctors. This study also shows substantial differences between the sampled doctors and their patients regarding treatment expectations and communication style (see Table 1), making the provision of long-term care to patients with OAB difficult at the primary care level. Lacking knowledge about OAB care and feeling embarrassed with patients also affected the confidence of the sampled doctors and their experiences in treating their patients. To overcome these problems, it is suggested that, through narrative and role modelling techniques [25], greater emphasis be placed on teaching medical students about the importance of humility [24]. Doing so may improve both the treatment and treatment provision experiences of patients and doctors. However, developing an awareness of humility and willingness to admit limitations in treating OAB may be challenging for doctors in Chinese cultures, in which doctors have become accustomed to high social status and deference, and thus a sense of arrogance among doctors may be inevitable. This also requires changing the stereotypes and expectations of doctors among the general public, which may prove difficult. Therefore, to enable primary care doctors to serve as gatekeepers in OAB care as suggested by the American Urological Association, it is crucial to enhance their knowledge about OAB through continuing medical education so that they are empowered in long-term care provision for patients with OAB and can preserve their professional dignity without breaking the taboo of admitting their limitations. Introducing empathetic communication skills and addressing the specific psychological needs of patients with OAB in undergraduate, postgraduate, and continuing medical education may yield improved experiences for both patients and doctors. These skills are particularly critical for primary care doctors because they are the first contact point for patients, and should aim to provide continual and comprehensive care to patients.

**Table 1:** Conflicting explanatory models between the sampled doctors and their patients with OAB

	Sampled doctors	Patients (as perceived by the sampled doctors)
Perception on the patients’ bladder complaints	1. Chronic condition	1. Infection due to rare bacteria
Expectations of treatment	1. Consultation and physical examination alone are considered as treatment 2. Prescribing medication is not necessary	1. Being prescribed with medication is a norm; merely consultation and physical examination are not enough to accomplish a treatment 2. Expect antibiotics in some cases
Expected outcome	1. Limitations to confirm OAB in primary care setting 2. Full recovery as impossible since OAB is a chronic condition	1. Expect definite diagnosis from doctors 2. Expect quick recovery 3. Not aware of the treatment limitations
When failing to experience improvement	1. Patience is required to see the improvement because of the limitations of current treatment 2. Suggest referral	1. Blamed doctors as incapable, deceptive money-making, prolonging treatment with bad intention to extract more money 2. Refuse referral
Communication style	1. Adopted casual communication style to ease patients’ embarrassment	1. Doctors were expected to show sincerity and empathy; casual communication style was perceived as unacceptable
History taking	1. Norm to ask for every possibility	1. Felt offensive when asked about their sexual life and substance abuse habit

### Limitations

This article summarises the experiences of the sampled doctors in treating patients with OAB in Hong Kong, thus the findings are specific to Hong Kong. Although performing a cross-comparison with primary care providers who have treated patients with OAB in other cultures is difficult because of the paucity of foreign literature on this topic, the current findings still provide an initial understanding that may be generalised to other cultures. The findings of this article were based on a sample of 30 primary care doctors working in the private sector in Hong Kong. Primary care doctors working in the public sector were excluded. Further research using larger samples with more therapeutic settings may provide a more holistic understanding of primary care doctor experiences in treating patients with OAB. Furthermore, this study was conducted by a single researcher. All the research procedures, including study conception and design, data collection, data analysis, and the writing of this article, were conducted by the researcher. Although data collection conducted by a single researcher ensured interview quality and consistency, it rendered the crosschecking of the entire study with other researchers impossible. To overcome the limitations raised by the participation of a single researcher, recoding of the transcripts was performed 1 month after the initial coding, which enabled the crosschecking of the analysed data, and to ensure that the codings and categories were free of ambiguity and overlap.

## Conclusion

This study investigated the treatment provision experiences of private practice primary care doctors treating patients with OAB. Lacking confidence in treating patients with OAB, experiencing mismatch with patients in both treatment expectations and communication style, and feeling embarrassed when communicating with OAB patients were the most common experiences encountered by the sampled doctors. The treatment provision experiences revealed a general lack of knowledge of OAB among the primary care doctors, thus affecting the extent of support that they could provide their patients with OAB. Furthermore, the negative stereotype of and lack of trust in private practice doctors created tension between the doctors and patients. This lack of mutual trust was particularly unfavourable for the doctors to provide long-term treatment and support to patients with OAB, whose bladder conditions are chronic and require long-term follow-up. The embedded distrust of private practice doctors also affected the prescribing behaviour of the doctors, who prescribed medication only to satisfy patient demands, which may lead to antibiotic abuse and resistance. Finally, the expectations of doctor professionalism and behaviour in Chinese cultures and the cultural perceptions of urinary diseases caused challenging treatment provision experiences for the sampled doctors. Without overcoming these challenges, primary care doctors in Hong Kong face difficulty in serving as gatekeepers of OAB care in the manner recommended by the American Urological Association.
